# Exosomes Derived from Human Primed Mesenchymal Stem Cells Induce Mitosis and Potentiate Growth Factor Secretion

**DOI:** 10.1089/scd.2018.0200

**Published:** 2019-03-08

**Authors:** Oliver Yuan, Clayton Lin, Joseph Wagner, Joehleen A. Archard, Peter Deng, Julian Halmai, Gerhard Bauer, Kyle D. Fink, Brian Fury, Nicholas H. Perotti, Jon E. Walker, Kari Pollock, Michelle Apperson, Janelle Butters, Peter Belafsky, D. Gregory Farwell, Maggie Kuhn, Jan Nolta, Johnathon D. Anderson

**Affiliations:** ^1^ Department of Otolaryngology, University of California, Davis, Davis, California.; ^2^ Drug Discovery Consortium, University of California, San Francisco, San Francisco, California.; ^3^ Department of Neurology, University of California, Davis, Davis, California.; ^4^ Good Manufacturing Practice Facility, University of California, Davis, Davis, California.; ^5^ Stem Cell Program, University of California, Davis, Davis, California.

**Keywords:** mesenchymal stem cells, exosomes, proteomics, HiRIEF LC-MS/MS, proliferation, extracellular matrix

## Abstract

Mesenchymal stem cells (MSCs) facilitate functional recovery in numerous animal models of inflammatory and ischemic tissue-related diseases with a growing body of research suggesting that exosomes mediate many of these therapeutic effects. It remains unclear, however, which types of proteins are packaged into exosomes compared with the cells from which they are derived. In this study, using comprehensive proteomic analysis, we demonstrated that human primed MSCs secrete exosomes (pMEX) that are packaged with markedly higher fractions of specific protein subclasses compared with their cells of origin, indicating regulation of their contents. Notably, we found that pMEX are also packaged with substantially elevated levels of extracellular-associated proteins. Fibronectin was the most abundant protein detected, and data established that fibronectin mediates the mitogenic properties of pMEX. In addition, treatment of SHSY5Y cells with pMEX induced the secretion of growth factors known to possess mitogenic and neurotrophic properties. Taken together, our comprehensive analysis indicates that pMEX are packaged with specific protein subtypes, which may provide a molecular basis for their distinct functional properties.

## Introduction

Numerous preclinical studies have demonstrated that mesenchymal stem cells (MSCs) hold promise as cell-based therapeutics for the treatment of inflammation-associated diseases [1–23]. The molecular mechanisms underlying MSCs' therapeutic properties remain inadequately characterized. Most published reports to date have focused on MSC-derived canonical secretory proteins as key drivers of functional recovery in animal models [24–30]. However, recent work from our laboratory and others have demonstrated that small, cellularly secreted vesicles called exosomes mediate much of MSCs' tissue healing effects, with administration of isolated exosomes capable of recapitulating many of the therapeutic effects observed via MSC transplantation [18,19,31–58].

Exosomes represent a recently characterized cell-to-cell communication system that transport numerous factors previously thought to be cell autonomous: nonsecretory proteins, RNAs, lipids, and metabolites [57,59–62]. Previous reports have focused on the RNA content of exosomes; however, our group and others have observed exosomal preps with substantially more protein content than RNA content, suggesting that the protein contents of exosomes may warrant further investigation.

The majority of published reports to date that have investigated the secretome of MSCs have done so using canonical expansion of cell culture conditions. However, the microenvironment experience by MSC postadministration into animal models and patients is strikingly different, including a substantial reduction in oxygen tension. Standard MSC culture conditions utilize an atmospheric oxygen tension of 20.95%, whereas various tissue compartments in the body can range from 1% to 5% O_2_. In addition, MSCs are generally culture using high levels of fetal bovine serum (FBS), which contains an abundance of embryonic growth-promoting factors. Such embryonic-associated paracrine and endocrine signaling factors are present in much lower concentrations in most adult tissues. Consequently, we have focused our efforts on understanding the secretome profile of MSCs transiently exposed to a more in vivo-like culturing system, using 1% O_2_ and serum deprivation to better model a more clinically relevant microenvironment.

Previously, we demonstrated that MSCs primed with such culture conditions increase expression of glycolytic, trophic, and mitogenic proteins, which were also reflected in the proteome of exosomes isolated from such primed MSCs (pMEX) [31].

However, it is currently unclear whether the proteins packaged into exosomes are done so in a stochastic manner or whether such packaging is a more regulated cellular process. The aim of this study was to assess whether pMEX are broadly enriched for specific classes of proteins, as this remains an outstanding question in the field. Toward this end, we compared the proteomic profiles of pMSC and pMEX, which revealed that pMEX are highly enriched with specific subclassifications of proteins, including secretory and extracellular matrix (ECM)-associated proteins. MSCs and their derived exosomes have been shown promise in preclinical studies for the treatment of diseases of the central nervous, increasing the plasticity of the effect neuronal tissue. The SHSY6Y cell line has been historically used for in vitro neuronal assays, based on their ability to differentiate from a progenitor cell phenotype to that of a mature neuronal phenotype. In this study, we demonstrated that pMEX are readily taken up by such neuroblast-like cells (SHSY5Ys) within 1 h of exposure and induce their cellular proliferation, as predicted based on the bioinformatic analysis of pMEXs proteome. We further determined that the most abundant protein packaged into pMEX was the ECM-associated protein, fibronectin, which, in part, mediated pMEXs mitogenic properties in cells of central nervous system (CNS) lineage.

## Materials and Methods

### Cell culture and exosome isolation

Five fresh human bone marrow aspirates were purchased from Lonza (Allendale, NJ). Human bone marrow is withdrawn from bilateral punctures of the posterior iliac crests of normal volunteers. Each donor is between the ages of 18 and 45 years and tested by Lonza and found to be nonreactive by an FDA-approved method for the presence of HIV-I, hepatitis B virus, and hepatitis C virus. After getting acceptable vital signs and hematology values, all donors are screened for general health and negative medical history for heart disease, kidney disease, liver disease, cancer, epilepsy, and blood or bleeding disorders. The Lonza Donor Program is currently approved, has been approved for over 10 years, and is submitted for annual approval by a commercial Institutional Review Board. MSCs were isolated from each donor via the established method of differential plastic adherence and use of MSC-media and maintained as separate cell lines. MSCs were isolated and then expanded by passing bone marrow aspirates through 90 μm pore cell strainers to isolate bone spicules. The strained aspirates were then diluted with an equal volume of phosphate-buffered saline (PBS) and centrifuged over Ficoll (GE Healthcare, Waukesha, WI) for 30 min at 700*g*. Then, mononuclear cells and bone spicules were plated in plastic tissue culture flasks, using minimum essential media α (MEM-α) (HyClone Thermo Scientific, Waltham, MA) supplemented with 10% premium select fetal bovine serum (FBS; Atlanta Biologicals, Lawrenceville, GA) that had been prescreened for optimal MSC growth. Following 2 days in culture, nonadherent cells were removed via three PBS washes. After the second passage, cells were expanded in 20% FBS in MEM-α, 1% l-Glutamine, and 1%Pen-Strep (MSC-media). Eligibility criteria for future studies were MSC populations that were 90% for the canonical MSC surface markers by passage 3 (CD73, CD90, and CD105) as assessed by flow cytometry evaluation using validated primary fluorochrome-conjugated monoclonal antibodies. MSCs expanded and cryopreserved until passages 6 before the initiation of use in studies. For pMEX isolation, MSCs were thawed and spun down (500*g*, 5 min) in 15 mL of full MSC media to eliminate cryopreservation reagent, dimethyl sulfoxide. MSCs were resuspended in MSC media and plated at a seeding density of 500–1,000 cells/cm^2^ in 30 mL of MSC media in T175 vented-cap flasks. Once cells reached 70% confluency, MSC flasks (∼100 × T175's per exosome isolation) were washed three times with 10 mL of PBS and subsequently placed in OptiMEM without phenol red with 1% l-Glut (IC) (Life Technologies, Carlsbad, CA) and exposed to 1% oxygen tension for 48 h. The resulting 3 L of conditioned media was processed for pMEX isolation using a preclearing centrifugation step at 1,000*g* for 15 min, followed by vacuum-assisted filtration using a 0.2 μM PES filter. The resulting solution was then ultrafiltered using tangential flow filtration with a molecular weight cutoff PES membrane of 100 kDa. Once concentrated, the pMEX solution is then diafiltrated using PBS to perform a buffer exchange using the same tangential flow filtration cartridge. pMEX protein concentration was determined using DC assay (Bio-Rad, Hercules, CA), and size distribution of vesicle diameter was determined using NanoSight LM10HS (Malvern, Amesbury, MA).

### Exosome uptake and proliferation

For uptake studies, pMEX were labeled with CellMask Green (Thermo Fisher, Carlsbad, CA) according to manufacturer's instructions. Negative controls consisted of an equal volume of PBS that was processed with either PKH26 or CellMask Green according to manufacturer's instruction. SHYSY's were plated into a six-well format tissue culture plate at 15,000 cells/cm^2^ and allowed to sit down overnight in 20% FBS in MEM-α, 1% l-glutamine, and 1%Pen-Strep. The following morning, the cells were washed three times with PBS before addition of OptiMEM without phenol red with 1% l-glutamine containing labeled pMEX or an equal volume of “labeled” PBS. One hour following exposure to treatment conditions, cells were washed three times with PBS and lifted with TrypLE for analysis via fluorescent microscopy or flow cytometry (Attune NxT; Thermo Fisher).

For proliferation studies, SHYSY5Y's were seeded at 9,000 cells/cm^2^ in a six-well format tissue grade plate, and expanded in 20% FBS in MEM-α, 1% l-glutamine, and 1%Pen-Strep. SHYSY5Y's were serum starved using Minimum Essential Medium Eagle alpha with 1% l-glutamine (Life Technologies) for 24 h after being washed three times with PBS. Following 24 h of serum deprivation, fresh serum media was placed on all cells with appropriate treatment condition using a six-well format tissue culture plate. Cells were incubated with pMEX then lifted with TrypLE. Cells were then evaluated for proliferation rates using CCK-8 assay (colorimetric assay) or Edu-FITC assay (flow cytometry) or nuclear staining with Hoechst 33342 (fluorescence microscopy). For inhibitor studies, cells were exposed to 100 μM of R-G-D-S peptide direct inhibitor of fibronectin binding or 10 μM of PHT427 (a pleckstrin homology domain, small molecule inhibitor to AKT) with or without 100 μg of pMEX before mitotic assessment. For growth factor secretion assessment, supernatants from SHSY5Y proliferation studies (100 μg pMEX vs. PBS control) were analyzed using RayBiotech's Q1 Growth Factor Quantibody array according to manufacturer's instructions. Proliferation studies were performed three times to verify the reproducibility of the observed results.

### Electron microscopy

pMSC and PMEX samples (*n* = 3 biological replicates) were fixed and dehydrated before scanning electron microscopy image acquisition with a Philips XL30 TMP (FEI Company, Hillsboro, OR). Sputter Coater: Pelco Auto Sputter Coater SC-7 (Ted Pella, Inc., Redding, CA). Transmission electron microscopy images were acquired using Philips CM120 Biotwin Lens, 9 (FEI Company, www.fei.com), with 2.0% uranyl acetate staining using facilities at Electron Microscopy Laboratory, School of Medicine, University of California at Davis.

### Sample preparation for proteomics

pMSC and pMEX derived from three different biological bone marrow aspirate donors were cultured, isolated, and pelleted as previously described. Pellets were lysed with 25 mM 4-(2-hydroxyethyl)-1-piperazineethanesulfonic acid (HEPES), 4% sodium dodecyl sulfate (SDS), and 1 mM dithiothreitol (DTT). Lysates were incubated at 95°C for 5 min then sonicated for 1 min, and centrifugation at 14,000*g* for 15 min. The supernatant was mixed with 8 M urea, 1 mM DTT, and 25 mM HEPES, pH 7.6, and transferred to a filtering unit with a 10 kDa cutoff (Nanosep^®^; Pall, Port Washington, NY), and centrifuged for 15 min at 14,000*g*, followed by another addition of urea buffer and centrifugation. Lysates were alkylated with 50 mM indoleacetic acid (IAA), 8 M urea, and 25 mM HEPES for 10 min and then centrifuged for 15 min at 14,000*g*, followed by two more additions of urea buffer and centrifugations. Trypsin (Promega, Madison WI) was added to lysates at a 1:50 trypsin:protein ratio and incubated overnight at 37°C. The lysate containing filters were then centrifuged for 15 min at 14,000*g*, followed by another centrifugation with MilliQ water and the flow-through was collected [63]. Peptides from pMSC and pMEX were labeled with tandem mass tag (TMT) TMT10 and TMT6, respectively, according to manufacturer's instructions (Thermo Fisher Scientific, San Jose, CA). Peptides were then cleaned by a strata-X-C-cartridge (Phenomenex, Torrance, CA) [63,64].

### Proteomics on nLC-MS/MS on thermo scientific LTQ Orbitrap Velos

Before analysis of pMEXs on LTQ-Orbitrap Velos (Thermo Fisher, San Jose, CA), peptides were separated using the Agilent 1200 nano-LC system. pMEX samples were trapped on a Zorbax 300SB-C18 and separated on a NTCC-360/100-5-153 (Nikkyo Technos, Tokyo, Japan) column with a gradient of “A” (5% dimethyl sulfoxide, 0.1% formic acid) and “B” (90% acetonitrile, 5% DMSO, 0.1% formic acid), ranging from 3% to 40% of “B” in 45 min with a flow of 0.4 μL/min. LTQ-Orbitrap Velos was operated in a data-dependent manner, which selected five precursors for the sequential fragmentation by collision-induced dissociation and higher energy collisional dissociation, and subsequently analyzed by the linear iontrap and orbitrap. The survey scan was completed in the orbitrap at 30,000 resolution from 300 to 2,000 m/z with a maximum injection time of 500 ms with automatic gain control set to 1 × 10^6^ ions. Generation of the higher energy collisional dissociation fragmentation spectra, a max ion injection time of 500 ms, and an automatic gain control of 5 × 10^4^ were used before fragmentation at 37.5% normalized collision energy. The normal mass range was used for Fourier transform mass spectrometry MS2 spectra, while centroiding the data at 7500 resolution. pMEX peptides for collision-induced dissociation were accumulated for a max ion injection time of 200 ms and of automatic gain control × 10^4^, fragmented with 35% collision energy, with the wideband activation on and activation of q 0.25 and an activation time of 10 ms before analysis at the normal scan rate and mass range in the linear iontrap. Precursors were subsequently isolated with a width of 2 m/z and positioned on the exclusion list for 60 s with both unassigned and single charge states being rejected from precursor selection.

### Proteomic data analysis

Panther Pathway analysis was used to detect the number of pathways detected in each sample and the number of proteins of each pathway represented in each sample (www.pantherdb.com). Ingenuity Pathway Analysis (IPA) software was used to analyze enrichment for signaling pathway proteins and putative functionality of proteins present in and between each sample with a significance threshold of 1% false discovery rate (Qiagen, Redwood City, CA www.ingenuity.com). ClueGO software was used for gene ontology and WikiPathway analysis of each sample to determine functionality of broad classes of proteins (www.ici.upmc.fr/cluego/). UniProt and IPA databases were also used for protein classification schemes.

### Statistical analyses

All statistical analyses were performed with GraphPad Prism V6.07. Where appropriate, *T* tests or multiple *T* tests with multiple testing correction were used with a false discovery rate of 1%.

## Results

### pMEX have canonical biophysical properties and co-isolate with FBS contaminants

**FIG. 1.**
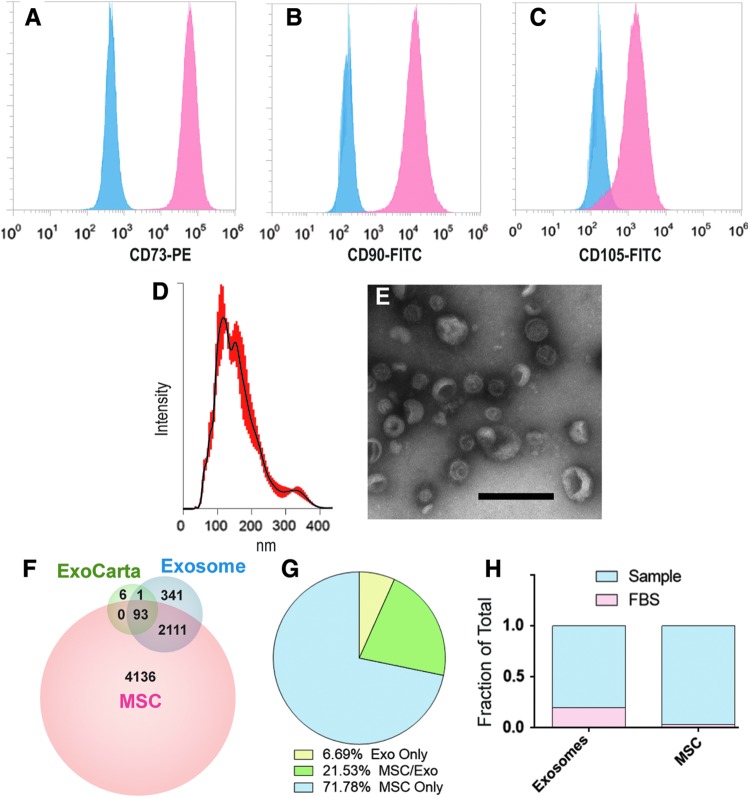
Flow cytometry, HiRIEF LC-MS/MS proteomics, nanoparticle tracking analysis and electron microscopy analysis of pMSCs and pMEXs. **(A–C)** Flow cytometry analysis of MSC surface marker expression using monoclonal primary conjugated antibodies against the canonical MSC markers CD73, CD90, and CD105. **(D)** Nanoparticle tracking analysis determined the size distribution of pMEX, with a mean diameter of 163 nm, *red highlight* = distribution of events, *black line* = median. **(E)** Transmission electron microscopy of pMEX with uranyl acetate negative staining (scale bar 200 nm). **(F)** HiRIEF LC-MS/MS proteomic analysis identified 93 and 94 exosomal markers out of the top 100 most cited in the ExoCarta database in pMSCs and pMEXs, respectively. **(G)** Of all, 6.7% proteins observed in pMEX were exclusively detected within pMEX, whereas 71.8% of all pMSCs proteins were exclusively detected in pMSCs. **(H)** FBS-derived proteins were detected in both pMSC and pMEX (*n* = 3/group, FDR 1%). FBS, fetal bovine serum; pMSCs, primed mesenchymal stem cells.

MSCs were isolated from human bone marrow purchased from Lonza, as previously described. After, passage 3 cells were assessed for expression of canonical MSC surface markers using flow cytometry analysis. MSC were over 90% for all three markers: CD73, CD90, and CD105 (Fig. 1A–C). Nanoparticle tracking analysis determined that pMEX possess a canonical diameter size distribution, with a mean diameter of 163 nm (*n* = 3 donors) (Fig. 1D). Transmission electron microscopy in combination with contrast staining demonstrated that pMEX have canonical exosome morphology as previously reported (Fig. 1E). Analysis of pMEX's tandem mass spectrometry proteomic profile established that they are packaged with 93 of the 100 most cited exosomal markers according to the ExoCarta database (ExoCarta.org) (Fig. 1F). Of all proteins detected across both pMSC and pMEX, 7% of proteins were exclusively detected in the pMEX samples (Fig. 1G).

pMSCs are initially passaged in expansion media up to passage 6, followed by three stringent washes with PBS before initiation of priming conditions (1% hypoxia and serum deprivation) for 48 h, the resulting conditioned media of which was harvested for pMEX. Ostensibly, such wash steps and serum-free priming conditions should limit the likelihood of co-isolation of bovine contaminants derived from FBS. However, we calculated the sum total of peak intensities for bovine proteins detected in both pMSC as well as pMEX and determined that pMSC contained ∼3% bovine material whereas pMEX contained a significantly higher ∼18% FBS-associated proteins (*n* = 3 donors/group, *P* < 0.01) (Fig. 1H). Therefore, better characterization of FBS contaminants of exosomal preps is warranted, especially when extracellular vesicles are isolated from serum containing isolation media, due to the increased risk of co-isolation bovine contaminants.

### pMEX contain elevated levels of specific subclassifications of proteins with distinct molecular functions

Although several studies have partially characterized the contents of exosomes, it remains unclear if exosomes are packaged with elevated levels of specific protein subtypes [36,50,59,65–72]. To this end, we comprehensively evaluated the proteomic profile of both pMSCs and pMEX based on protein localization and classification analysis using the IPA database. We assessed both, protein area (ie, relative mass) and the unique number of proteins associated with each category. This method helped elucidate whether a relatively large mass of a protein subtype is composed of just a few highly abundant proteins, or conversely, composed of numerous unique proteins present at low levels (ie, relatively low mass).

**FIG. 2.**
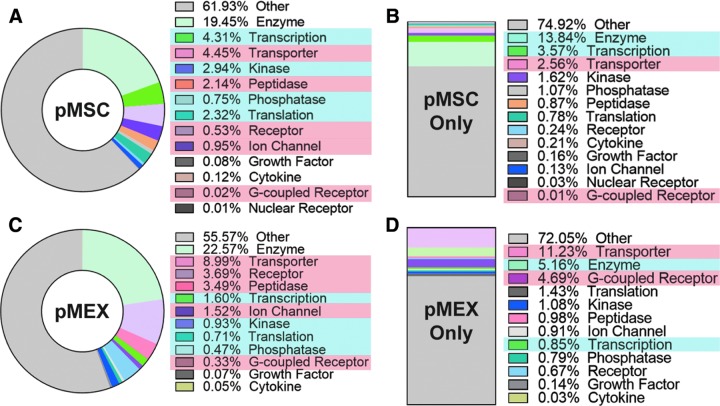
pMEXs are enriched for specific functional classifications of proteins. **(A, C)** IPA determined that pMEXs contain elevated fractions of transporters, peptidases, receptors, G-coupled receptors, and ion channels compared with pMSCs. pMEX contained lower fractions of transcription, kinase activity, translation, and phosphatase proteins compared with pMSCs. Proteins exclusively detected in pMSCs **(B)** contained elevated fractions of enzymes and transcription-associated proteins than proteins detected exclusively in pMEX. **(D)** Proteins exclusively detected in pMEX contained higher fractions of transporters and G-coupled receptors. *n* = 3/group, FDR 1%. *Red highlight* = higher fraction in pMEX, *blue highlight* = lower fraction in pMEX. IPA, Ingenuity Pathway Analysis.

pMEXs were packaged with proportionally elevated levels of specific subclassifications of proteins compared with the pMSCs from which they were derived. IPA demonstrated that pMEX contained elevated levels of transporters, peptidases, receptors, G-coupled receptors, and ion channels compared with pMSC based on relative protein abundance (*n* = 3 donors/group, *P* < 0.001) (Fig. 2A–D). Our analysis further determined that pMEX contained a lower proportion of proteins associated with transcription, kinase activity, translation, and phosphatases compared with pMSC based on relative abundance (*n* = 3 donors/group, *P* < 0.01) (Fig. 2C). Of the proteins detected exclusively in pMEX, we determined that transporter and G-coupled receptor proteins were present in greater abundance than in pMSC (*n* = 3 donors/group, *P* < 0.01) (Fig. 2D). Of the proteins exclusively detected in pMSCs, enzymes and transcription-associated proteins were present in higher percentages (*n* = 3 donors/group, *P* < 0.01) (Fig. 2B). These data establish that pMEX are enriched for specific protein classifications, with >6× increase in relative abundance of receptors and a ∼2× increase in transporter proteins compared with pMSC.

### Extracellular and plasma membrane proteins are the most abundant protein classes packed within pMEX

**FIG. 3.**
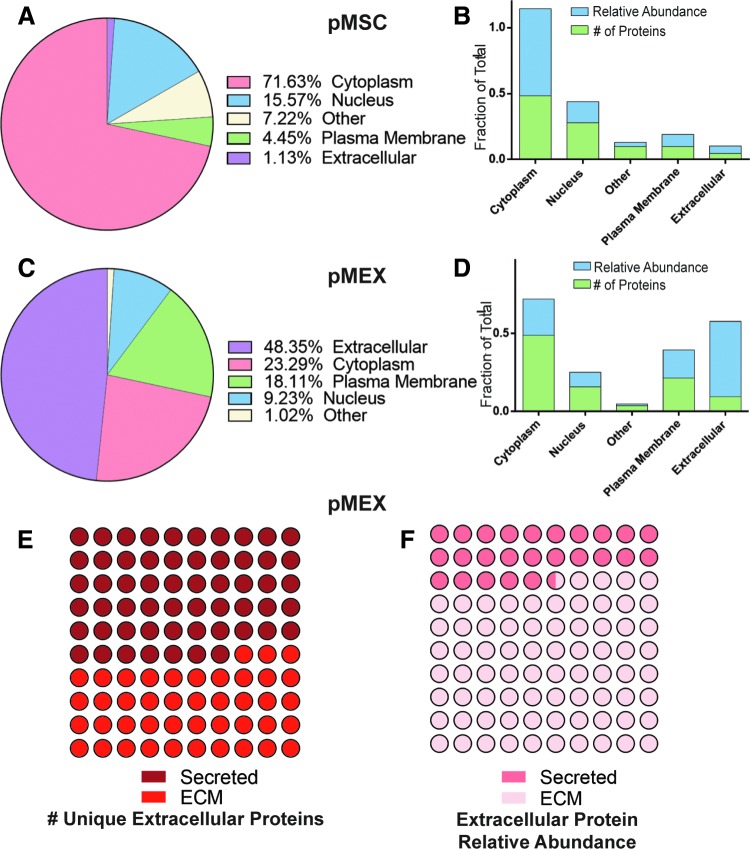
pMEXs are enriched for extracellular and plasma membrane proteins. **(A)** Distribution of all proteins across cellular sublocalization classes in pMSC and **(B)** proteins exclusively detected in pMSC according to IPA. **(C)** Distribution of all proteins across cellular sublocalization classes in pMEX, and **(D)** proteins exclusively detected in pMEX according to IPA. **(E)** Fraction of pMEX unique extracellular proteins that were either associated with ECM or secretory proteins. **(F)** Fraction of pMEX proteins abundance associated either with ECM or secretory proteins. *n* = 3/group, FDR 1%. ECM, extracellular matrix.

We analyzed our proteomics data to determine whether pMEX are packaged with elevated levels of proteins associated with a specific subcellular localization compared with their parental cell line, pMSCs. pMEX contained 4-fold and 42-fold increase in receptor and extracellular proteins than pMSC, respectively (*n* = 3 donors/group, *P* < 0.005) (Fig. 3A–D). Of all specific extracellular pMEX proteins, approximately half are ECM derived, however, these ECM proteins comprise about ∼74.5% of all extracellular pMEX proteins, based on relative abundance (Fig. 3E, F). Therefore, a relatively small number of ECM proteins comprise ∼35.8% of pMEXs total relative protein content.

pMEX are also packaged with 300% and 70% decrease of cytoplasmic and nuclear localized proteins, respectively, compared with pMSCs based on relative abundance (*n* = 3/group, *P* < 0.005) (Fig. 3A–D). Although pMEXs contained a similar fraction of specific cytoplasmic proteins as pMSCs, the relative abundance of these cytoplasmic proteins was substantially less in pMEXs (*n* = 3 donors/group, *P* < 0.005) (Fig. 3A–D). This indicates that the cytoplasmic proteins in the exosomes were generally present at much lower levels (ie, relative mass) than in pMSCs. We observed numerous proteins that were detected exclusively either in pMSCs or pMEXs. Of the proteins exclusively detected in pMEXs, there was 10× and 3× increase in extracellular and plasma membrane-associated proteins compared with proteins exclusively detected in pMSC based on relative mass (*n* = 3 donors/group, *P* < 0.005) (Fig. 3A–D). These data demonstrate that pMEX contained higher fractions of both extracellular and plasma membrane-associated proteins compared with pMSCs.

### pMEX are packaged with ECM proteins associated with proliferation

**FIG. 4.**
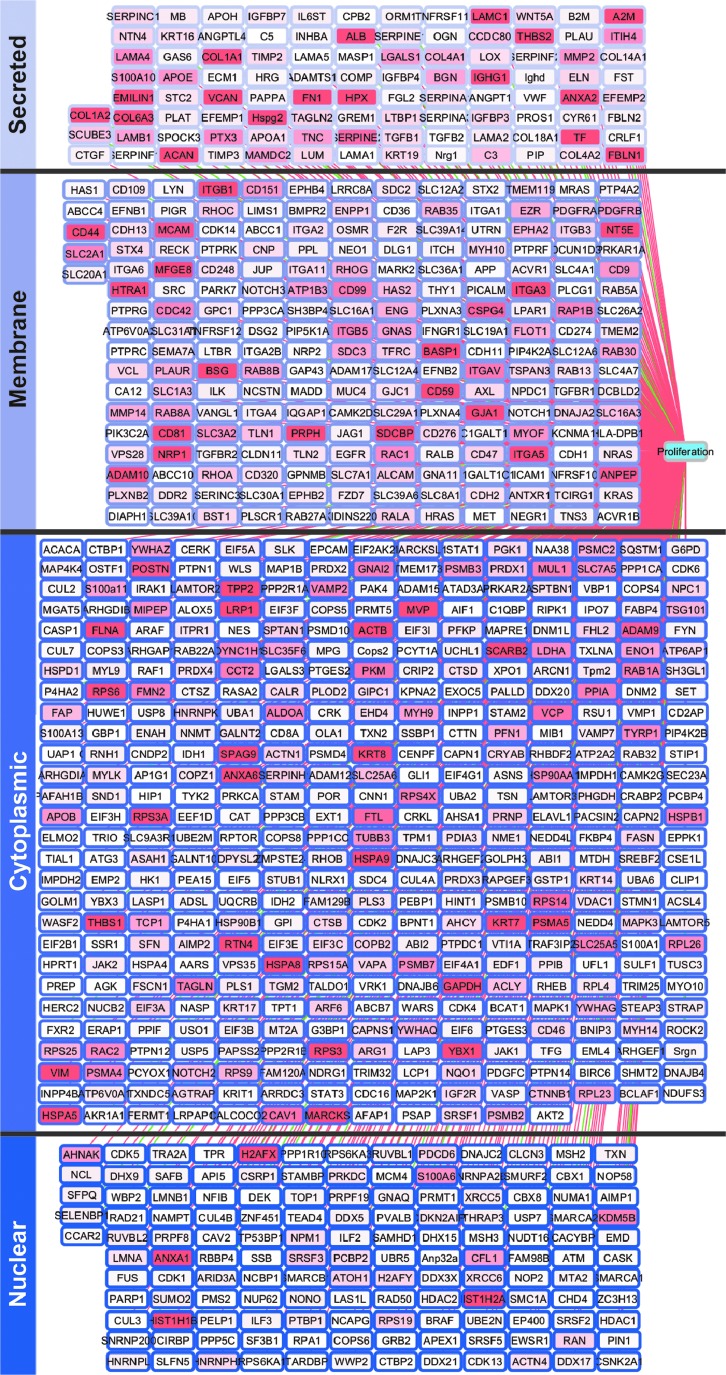
pMEXs are packaged with proliferation-associated proteins. IPA of pMEXs proteomic profile established presence of 701 mitogenic proteins. Relative abundance of each protein is indicated by the depth of shading of each node. *Pink edge lines* represent proteins known to induce proliferation, *green edge lines* represent proteins known to inhibit proliferation, *n* = 3, FDR1%.

Studies have established that MSCs have mitogenic properties. Therefore, we examined the proteomic profile of pMEX for genes associated with either the induction or inhibition of cellular proliferation. IPA demonstrated that pMEX are packaged with ∼700 proliferation-associated proteins, of which 689 were associated with increased proliferation (Fig. 4). Based solely on the number of unique individual proteins detected, cytoplasmic proteins were the largest represented protein class in pMEX (Supplementary Fig. S1). However, based on relative abundance, extracellular proteins were the most abundant class and accounted for 43% of the total proliferation-associated protein content detected in pMEX (Supplementary Fig. S1A, B). Enrichment of mitogenic extracellular proteins suggests that pMEXs proliferative properties may be mediated by such proteins.

### Extracellular protein fibronectin mediates pMEXs mitogenic properties

**FIG. 5.**
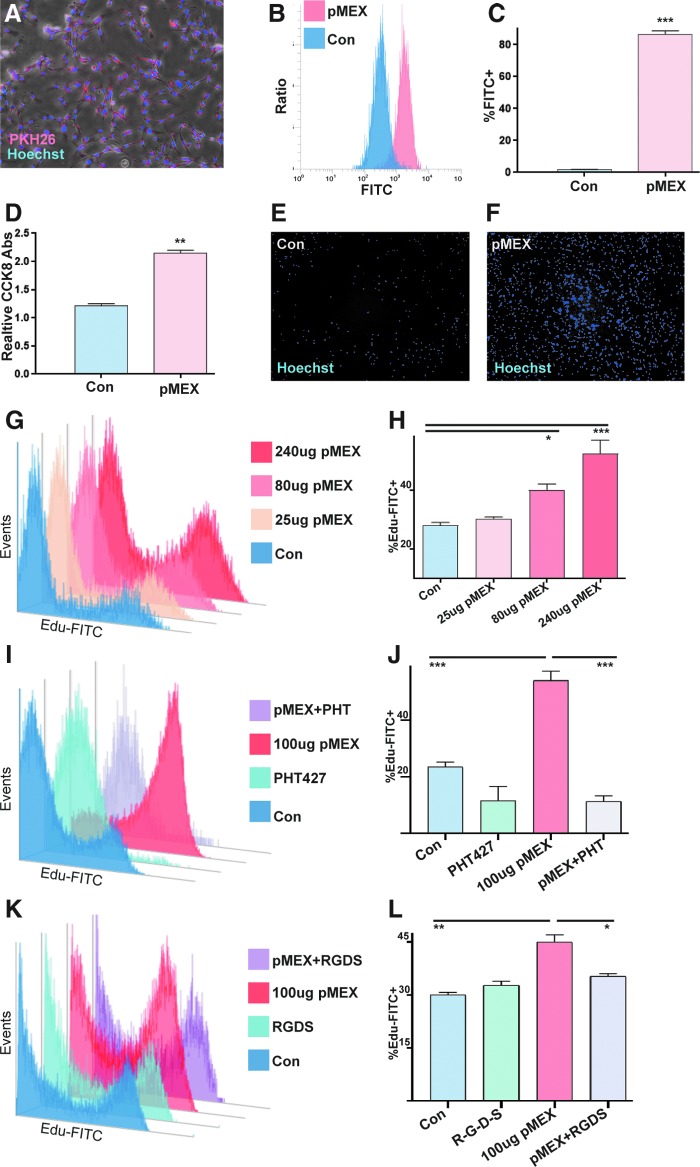
pMEX are readily taken up by cells and induce proliferation. **(A)** 100 μg pMEX were fluorescently labeled with the lipophilic dye, PKH26, washed, and then exposed to SHSY5Y cells for 1 h, and evaluated via fluorescent microscopy with a 4× objective. SHSY5Y cells costained with Hoechst 33342. **(B, C)** Quantification of pMEX uptake by SHSY5Y's was determined using flow cytometry analysis of SHSY5Y exposed to 100 μg CellMask Green fluorescent labeled pMEX for 1 h, compared to CellMask Green “labeled” PBS controls. **(D–F)** Treatment of serum-deprived SHSY5Y cells with 100 μg pMEX for 18 h induced proliferation as determined by CCK8 colorimetric assay followed by fluorescent microscopy of Hoechst 33342-stained cells. **(G, H)** Following 18 h serum deprivation, SHSY5Y's were treated with 100 μg pMEX and assessed for proliferation 18 h posttreatment with flow cytometry analysis of FITC-labeled Edu compared with vehicle (PBS) controls. **(I, J)** SHSY5Y's were treated with 100 μg pMEX or vehicle controls (PBS), in the presence or absence of 10 mM AKT-specific inhibitor (PHT427) and proliferation was evaluated using Edu-FITC assay. **(K, L)** SHSY5Y's were treated with 100 μg pMEX or vehicle control (PBS), in the presence or absence of 100 μM R-G-D-S peptide inhibitor and proliferation was assessed using Edu-FITC assay. All proliferation studies were performed three times to verify reproducibility *n* = 3, *T* test analysis was used to test for significance, *P < 0.05, **P < 0.01, ***P < 0.005. PBS, phosphate-buffered saline.

Next, we proceeded to investigate whether the abundant extracellular-associated proteins packaged into pMEX may mediate some of their functional properties. The observation of numerous proliferation-associated proteins detected in pMEX, many of which were extracellular in nature, led us to investigate the mitogenic capacity of pMEX. We determined that fluorescently labeled pMEX (100 μg) are taken up by the neuroblast-like cell line, SHSY5Y's, within 1 h of exposure by fluorescent microscopy and flow cytometry analysis (*n* = 3 replicates/group, *P* < 0.005) (Fig. 5A–C). Treatment with 100 μg pMEX significantly induced proliferation of SHYSY5 cells compared with vehicle (PBS)-treated controls, as determined by both CCK8 absorbance as well as image evaluation of Hoechst 33342 nuclear-stained cells (*n* = 3 replicates/group, *P* < 0.005) (Fig. 5D–F). Using the more sensitive Edu proliferation assay, we established that pMEX induced proliferation in a dose-dependent manner as evaluated via flow cytometry (*n* = 3 replicates/group, *P* < 0.005) (Fig. 5G, H).

Since extracellular proteins were the most abundant class of proliferative proteins in pMEX, we proceeded to investigate whether secreted proteins mediated pMEXs mitogenic properties. Fibronectin was determined to be the most overall abundant protein in pMEX, which provided the rationale for testing the inhibition of this pathway to determine if fibronectin signaling mediates pMEXs mitogenic properties. We used a specific inhibitor of AKT signaling (PHT427), which binds the pleckstrin homology domain of AKT, blocking its ability to phosphorylate proteins downstream in the fibronectin signaling cascade. Our data demonstrated that AKT inhibition (10 μM) significantly attenuated pMEX-induced proliferation in SHYSY5Y's (*n* = 3 replicates/group, *P* < 0.0005) (Fig. 5I, J). To validate the involvement of fibronectin signaling in the mitogenic properties of pMEX, we next used a competitive binding peptide inhibitor of fibronectin signaling (R-G-D-S) concurrent with 100 μg pMEX treatment in SHSY5Y cells. The R-G-D-S fibronectin inhibitor (100 μM) attenuated the mitogenic capacity of 100 μg pMEX treatment by 22% in the SHSY5Y cell line (*n* = 3 replicates/group, *P* < 0.05) (Fig. 5K, L). Collectively, these data demonstrated that pMEX are packaged with extracellular proteins which, in part, mediated their ability to potentiate cellular proliferation in a fibronectin-dependent manner. All proliferation studies were performed three times to verify reproducibility.

### pMEX potentiated secretion of growth factors by neuroblast-like cells

**FIG. 6.**
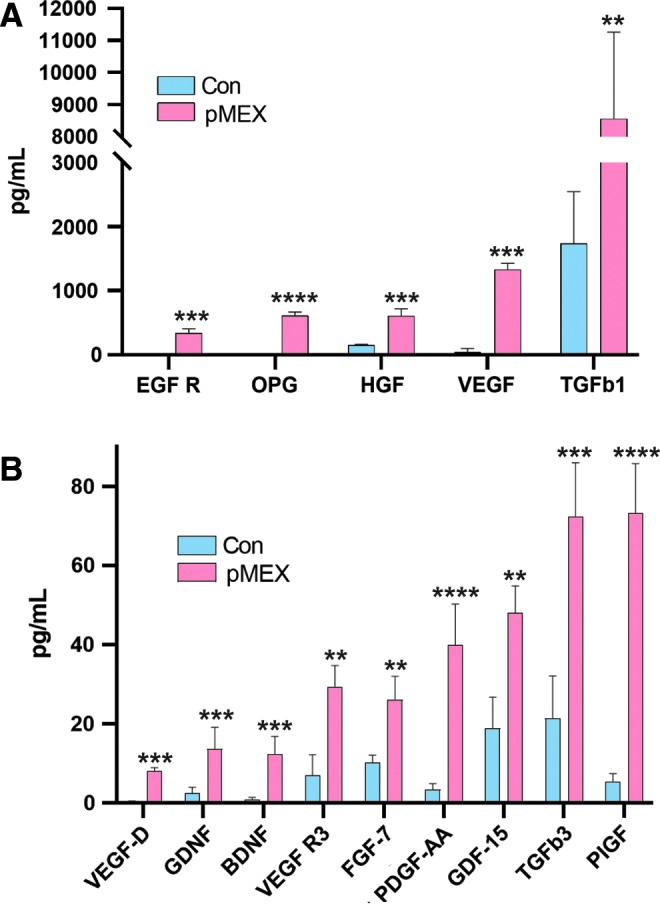
pMEX induce secretion of growth factors and neurotrophic proteins. **(A, B)** Following 18 h serum deprivation SHSY5Y cells were exposed to 100 μg of pMEX or PBS vehicle controls for 24 h before secretome analysis of the resulting conditioned media via multiplexed sandwich ELISA array (Quantibody). *n* = 4, multiple *T* tests with a false discovery rate of 1% was used to test for significance, **P < 0.01, ***P < 0.005, ****P < 0.001.

Next, we investigated whether 100 μg pMEX treatment modulated the secretory profile of SHSY5Y cells. We used a multiplexed sandwich ELISA cytokine array (Quantibody) (Supplementary Fig. S2) to quantitatively assess growth and trophic factor secretion of SHSY5Y's 24 h post-pMEX (100 μg) treatment. Multiplexed sandwich ELISA cytokine array analysis determined that pMEX treatment increased secretion of 14 factors with well-established proliferative and trophic properties (*n* = 4/group, *P* < 0.05–*P* < 0.0005) (Fig. 6A, B). These factors have been established as mediators of cellular proliferation and their increased secretion positively correlated with pMEXs mitogenic properties.

## Discussion

There is growing interest in MSC-derived exosomes both as a means to elucidate MSCs' mechanisms of action, and as potential standalone monotherapy. However, little is understood about the physiology of exosomes derived from MSCs. One outstanding question has been which factors are enriched in MSC-derived exosomes and what functional properties do they convey when their biogenesis is potentiated under physiological conditions [18,19,41–48,54,73–75]. In this study, we determined that the most abundant proteins detected in pMEXs were of an extracellular origin. We also observed a several fold enrichment of receptor and transporter proteins in pMEXs compared with the MSC parental lines from which they were derived. These data indicate that the most abundant exosomal proteins are extracellular and plasma membrane associated, which may have important implications for their observed functional properties.

We determined that pMEXs were packaged with numerous extracellular and plasma membrane-associated proteins predicted to induce proliferation. We functionally validated the mitogenic properties of pMEX using a cell line with neuronal properties (SHSY5Y), an indication that the specific proteins packaged into exosomes are predictive to some degree of their physiological properties. Interestingly, we found over 400 proteins detected exclusively in exosomes, although the lack of detection of these proteins in the MSCs may be attributable to masking effects due to their more complex cellular lysate. However, it is feasible that at least a subset of these proteins is expressed exclusively for secretion into exosomes. To date, most published reports of the functional properties of MSC-derived exosomes have isolated extracellular vesicles from canonical MSC culture media and oxygen tension. There remains the possibility that such conditions induce cellular signaling cascades that induce differences in the secretome of MSCs compared with MSCs cultured under conditions that more closely mimic those experienced by MSCs postadministration in vivo. Hence, further investigation into the potential differences between exosomes isolated under such different conditions may be informative to the field, both in terms of their proteomic packaging as well as their pleotropic functional properties.

## Conclusion

Taken together, the data from this investigation suggest that the packaging of exosomes with proteins is not performed in a stochastic manner, as we observed substantial enrichment of specific proteins and protein classes compared with their parental cell line. Further work is warranted to elucidate the mechanisms by which cells regulate protein packaging in exosomes. Although several preclinical studies support the application of MSC exosomes as a novel therapeutic platform, elucidating the mechanisms of action is critical in advancing this technology.

## Supplementary Material

Supplemental data

Supplemental data
