# Supplementary material for: Exosomes Derived from Human Primed Mesenchymal Stem Cells Induce Mitosis and Potentiate Growth Factor Secretion
Source: Stem Cells Dev. 2019 Mar 8;28(6):398–409. doi: 10.1089/scd.2018.0200 (PMC6441283; doi:10.1089/scd.2018.0200)
Supplement: Supplemental data [file Supp_Fig1.pdf]

Supplementary Data

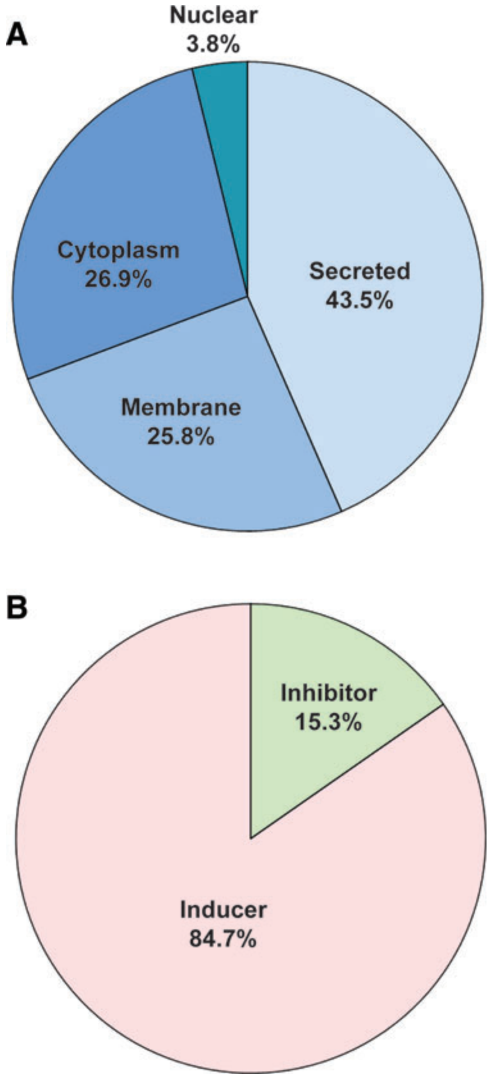

SUPPLEMENTARY FIG. S1. pMEX proliferation-associated proteins enriched for secretory proteins. **(A)** HiRIEF LC-MS/MS proteomic analysis followed by IPA analysis determined that the most relatively abundant subcellular classification of pMEX proteins was secretory in nature. **(B)** IPA analysis showing relative abundance distribution of pMEX proteins known to be inducers vs inhibitors of proliferation. $n = 3$, FDR1%. IPA, Ingenuity Pathway Analysis; pMEX, primed mesenchymal stem cells secrete exosomes.
