# Supplementary material for: Exosomes Derived from Human Primed Mesenchymal Stem Cells Induce Mitosis and Potentiate Growth Factor Secretion
Source: Stem Cells Dev. 2019 Mar 8;28(6):398–409. doi: 10.1089/scd.2018.0200 (PMC6441283; doi:10.1089/scd.2018.0200)
Supplement: Supplemental data [file Supp_Fig2.pdf]

AR	IGFBP-4
BDNF	IGFBP-6
bFGF	IGF-I
BMP-4	Insulin
BMP-5	MCSF R
BMP-7	NGF R
b-NGF	NT-3
EGF	NT-4
EGF R	OPG
EG-VEGF	PDGF-AA
FGF-4	PIGF
FGF-7	SCF
GDF-15	SCF R
GDNF	TGFa
GH	TGFb1
HB-EGF	TGFb3
HGF	VEGF
IGFBP-1	VEGF R2
IGFBP-2	VEGF R3
IGFBP-3	VEGF-D

SUPPLEMENTARY FIG. S2 Complete list of proteins assessed via Quantibody multiplexed sandwich ELISA array. *Grayed boxes* were not detected in the samples tested.
